# The influence of electrical high-speed rotation on mandibular third molar surgeries: a prospective, randomized, split-mouth clinical and radiographic study

**DOI:** 10.1038/s41598-024-59611-5

**Published:** 2024-04-17

**Authors:** Izabella Sol, Karen Rawen Tonini, Karen Santin dos Reis, Henrique Hadad, Daniela Ponzoni

**Affiliations:** 1 Department of Diagnosis and Surgery, Araçatuba School of Dentistry-UNESP, José Bonifácio no 1193, Araçatuba, SP 16015-050 Brazil; 2 Araçatuba School of Dentistry-UNESP, José Bonifácio no 1193, Araçatuba, SP 16015-050 Brazil

**Keywords:** Bone regeneration, Oral surgical procedures, Molar, third, Osteotomy, Tooth extraction, Dentistry, Dental equipment, Health care, Quality of life

## Abstract

The aim of this split-mouth randomized clinical trial was to evaluate the clinical outcomes (operative time, edema, trismus, and pain), the immediate histological effects, the alveolar repair (2 and 4 months), and the quality of life after the extraction of impacted third molars using high-speed pneumatic and electrical rotation. Sixteen patients underwent extraction of the two mandibular third molars with a minimum interval of 15 days. On one side of the participant’s mouth, high-speed pneumatic rotation was used (Control Group—CG) while for the other side, high-speed electrical rotation was used (Study Group—SG). Statistical analysis included ANOVA repeated measures and Pearson correlations. SG group showed: shorter operative time (p = 0.019), less pain (p = 0.034), swelling (p < 0.001) and trismus (p = 0.025) on the 1st postoperative day; less pain (p = 0.034) and trismus (p = 0.010) on the 3rd postoperative day; less trismus (p = 0.032) on the 7th postoperative day; and better quality of life (p = 0.007). No differences were observed for peripheral bone damage or bone density of alveolar repair at 2 and 4 months between groups. Electric high-speed rotation provided better postoperative clinical parameters of pain, edema and trismus when compared with pneumatic high-speed rotation for mandibular third molar surgery.

**Trial registration:** Brazilian Registry of Clinical Trials registration number RBR-4xyqhqm (https://ensaiosclinicos.gov.br/rg/RBR-4xyqhqm).

## Introduction

The extraction of impacted third molars is one of the most frequent treatments carried out by Oral and Maxillofacial Surgeons^1^. The use of panoramic radiographs to diagnose the presence of third molars helps in the planning of the surgical procedure^1,2^, and the classifications of Winter (1926) and Pell & Gregory (1933) are the most used for this purpose^1^. Depending on the degree of impaction, rotary cutting instruments may be required for osteotomy and odontosection procedures, which have been continuously improved to minimize the invasiveness of the procedure^3–7^.

Although studies have shown that rotary cutting instruments produce higher temperatures during osteotomy, which can lead to peripheral necrosis and impair bone repair^8,9^, pneumatic high-speed turbines are still the most commonly used instruments in outpatient surgeries^4,9^. They work at high speed (350,000–450,000 rpm), produce noise, vibration^10^, and have a low torque, losing speed when faced with obstacles^11^. Electric motors have replaced these turbines in dental equipment. They produce speeds of 50,000–200,000 rpm while maintaining the flexibility of a continuous high-torque drive system, enabling the operator greater tactile sensitivity^4,9,10^.

Although considered a relatively common procedure, the removal of impacted third molars is an invasive surgery. Postoperative pain, edema and trismus due to surgical trauma are expected complications^3,5^. Depending on the intensity of these complications, the patient’s routine may be affected. The assessment of the impact of third molar surgery on daily activities and on the patient’s general well-being is essential for clinical decision-making and adequate postoperative guidelines^12^. It is therefore important to search for new techniques to improve precision and surgical safety, which minimize postoperative complications and provide greater comfort in the post-extraction period^5,13^.

Studies in the areas of restorative and prosthetic dentistry have shown that electric motors have better cutting efficiency with less heat production when compared with pneumatic turbines^10,11^. However, in Oral and Maxillofacial Surgery, most of the studies are currently focused on piezosurgery^5–7,9,14^ which, despite causing less tissue trauma has a longer trans-surgical time^7^. There is a gap in the literature of studies in the surgical area on the effect of the electric motor in outpatient surgeries, and how it compares with other cutting systems^4^.

The aim of this randomized clinical study was to compare clinical parameters of pain, edema, trismus, quality of life, in addition to local bone repair and peripheral necrosis index after extraction of impacted lower third molars using a high-speed pneumatic and electric turbine. The null hypothesis of this study is that there would be no difference in clinical and radiographic parameters after extraction of third molars using two types of high-speed turbine systems.

## Materials and methods

### Study design

This was a randomized, prospective, “split-mouth” clinical study, developed at the São Paulo State University (Unesp), School of Dentistry, Araçatuba. The research project was approved by the Research Ethics Committee on 07/16/2021 (CAAE: 49101521.2.0000.5420), registered in the Brazilian Registry of Clinical Trials on 18/10/2022 (RBR-4xyqhqm) and followed the CONSORT 2010 guidelines. The study adheres to the tenets of Declaration of Helsinki. Informed consent was signed by each participant. The researchers involved had no conflict of interest.

**Figure 1:**
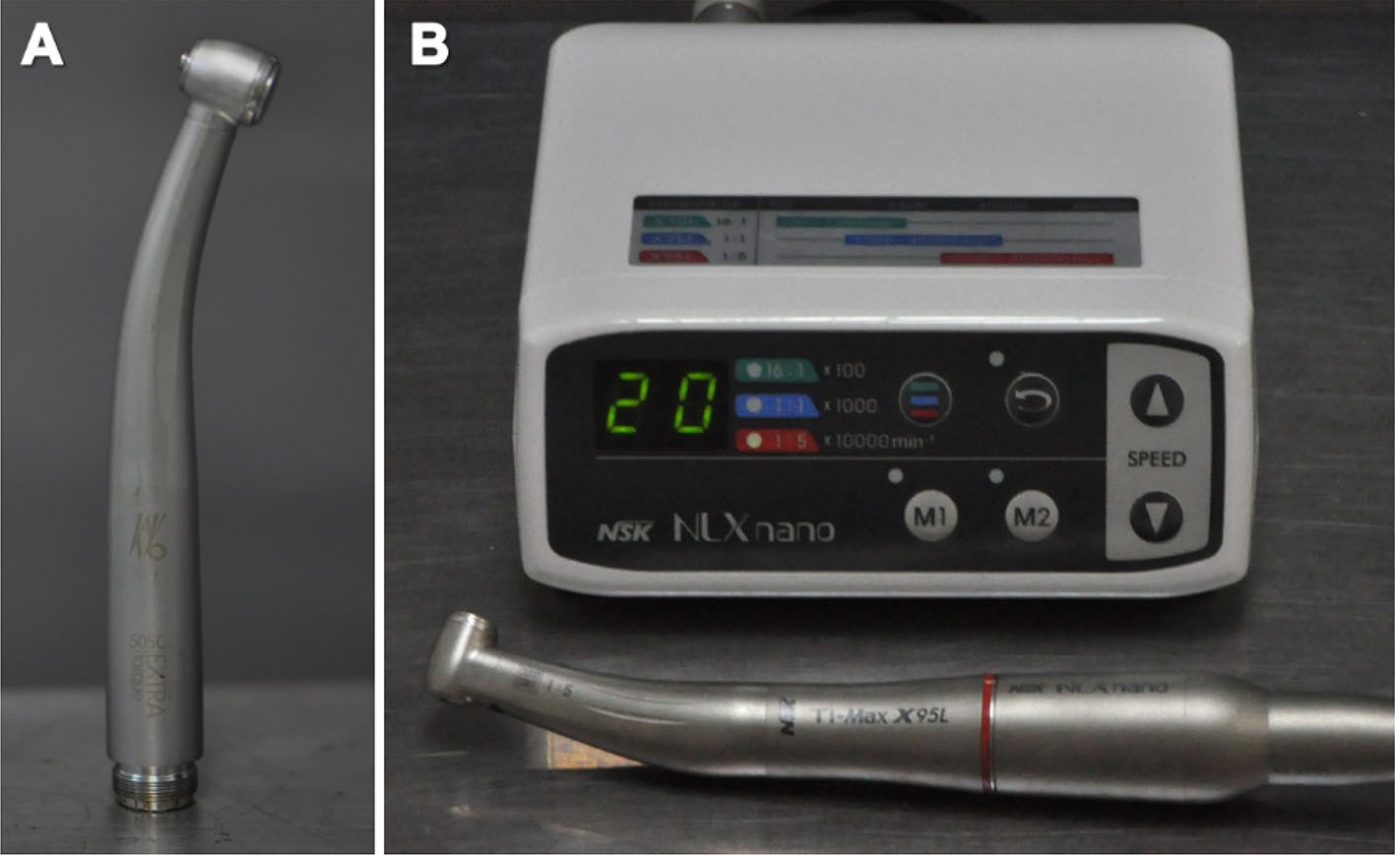
High-speed turbines used. (**A**) Pneumatic and (**B**) electric high-speed turbine.

The statistical power and sample size were calculated based on previous studies^6^, using the website http://www.biomath.info/power/prt.htm. A minimum sample size of n = 6 was obtained, for a statistical power of 80% in a one-tailed hypothesis, at a significance level of 5%, and a difference to be detected of 20.39. The patients selected for the extraction of the two mandibular third molars received both types of treatment, and were divided into two groups, according to the turbine used for the osteotomy and odontosection procedures: Control Group (CG) with the use of a pneumatic high-speed turbine (Kavo505c, Joinville, SC, Brazil) at 450,000 rpm; and Study Group (SG) with electric high-speed turbine with surgical multiplier 1:5 (NSK, Bauru, Brazil) at 200,000 rpm (Fig. 1).

Participants should have good systemic health, aged between 15 and 25 years, who had both impacted mandibular third molars, completely or partially unerupted, in similar positions, whose panoramic radiographs images suggested that their removal would demand osteotomy and odontosection were included in the study^9,11^. The teeth were classified according to the Pell & Gregory classification into B/C, II/III and Winter positions (mesioangular, distoangular, horizontal or vertical)^3^.

Patients who had extensively decayed third molars, with periodontal disease, patients who use alcohol and/or drugs, smokers, patients with systemic disorders, allergic to penicillin, and pregnant or nursing women were excluded^8,9,11^.

The teeth were removed in two surgical sessions, with a minimum interval of 15 days, by the same operator^9,11^. For each patient, one tooth was randomly included in the CG group and another in the SG group and the patient was blinded to that process. The allocation was made by a third researcher using two envelopes containing the word pneumatic and electrical inside each one^11^. Due to the obvious differences of the turbines under study, blinding the operator was not possible.

### Surgical procedure

All patients were prophylactically medicated with 1 g of Amoxicillin and 600 mg of Ibuprofen, 1 h before the procedure^6^. After intraoral and extraoral antisepsis with 0.12% aqueous chlorhexidine digluconate, patients were anesthetized with 2% mepivacaine with 1:100,000 epinephrine (Mepiadre, DFL, Rio de Janeiro, Brazil) using inferior alveolar, buccal and lingual nerve blocks. An envelope incision was made with a nº 15 scalpel blade, and total mucoperiosteal detachment with a nº 9 Molt detacher was carried out.

Afterwards, buccal and distal osteotomies and odontosections were performed with a 702 long stem conical drill under irrigation with sterile saline solution. In the CG group, a high-speed pneumatic turbine was used (Kavo505c, Joinville, Brazil) and in the SG group, a high-speed electric turbine coupled to a 1:5 surgical multiplier was used (NSK, Bauru, Brazil). Extraction was performed with the aid of dental extractors.

**Figure 2:**
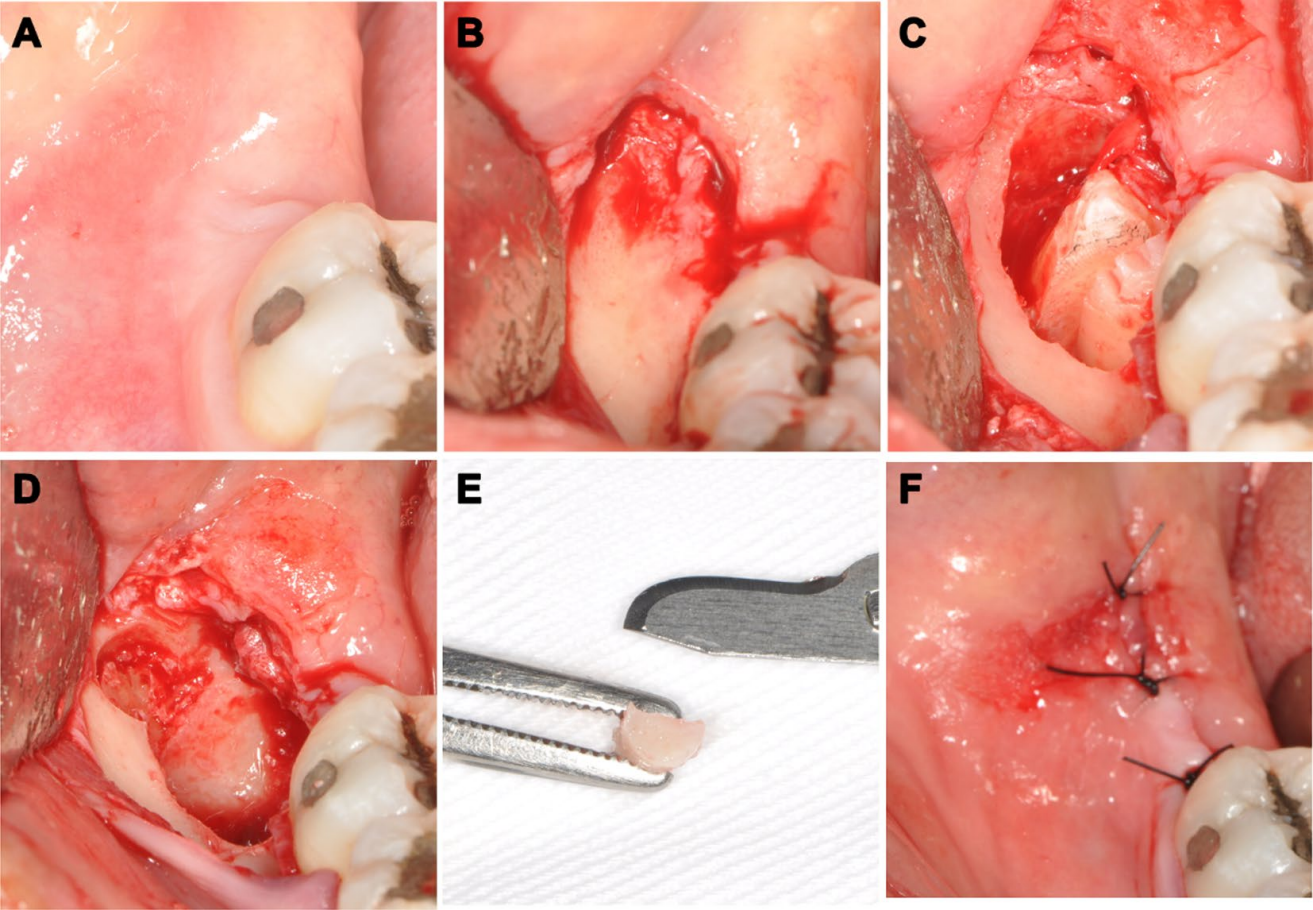
Surgical sequence for both groups. (**A**) Initial appearance. (**B**) Envelope incision and mucoperiosteal detachment. (**C**) Osteotomy and odontosection. (**D**) Aspect of the socket after tooth extraction and distal biopsy. (**E**) Distal biopsy of the socket. (**F**) Sutures.

Peripheral bone was collected using a 3 mm diameter trephine (WF Cirúrgicos, Barueri, Brazil) in the distal region of the bone socket and stored in 10% formaldehyde for analysis of the immediate bone necrosis index^4^ and bone regularity^4,7,15^. The sockets were irrigated with saline solution, filled with clot, and sutured using interrupted stitches with 4.0 nylon thread (Procare, Itajai, Brazil) (Fig. 2).

The operative time of each extraction was measured in minutes with the aid of a digital stopwatch, starting at the time of the incision and ending at the end of the suture.

Postoperative medication prescribed was 500 mg amoxicillin 3 times a day for 7 days, 300 mg ibuprofen 3 times a day for 3 days, dipyrone 1 g 4 times a day for 2 days, and mouthwash with 15 ml 0.12% chlorhexidine digluconate twice a day for 7 days^3,6^. The stitches were removed after 7 days.

### Variables analysis

Data were organized into primary and secondary outcomes. The primary outcomes of this clinical study were represented by the parameters of pain, edema and trismus evaluated on the 1st, 3rd and 7th postoperative day^5,7^. Secondary outcomes were represented by the immediate necrosis range, alveolar repair (assessed by panoramic radiography after 2 and 4 months) and the quality of life questionnaire assessment on the 7th postoperative day. The confounding variables evaluated were age, gender, surgical time and dental classification according to Pell & Gregory and Winter.

Postoperative pain was assessed using a visual analogue scale (VAS) with a 0–10 unit scale graph (0 for no pain, 1–3 for mild pain, 4–6 for moderate pain, 7–9 for severe, and 10 for excruciating pain)^3,7,8,16,17^. For data analysis, each score received a new score to facilitate statistical analysis. Thus, variations 0, 1–3, 4–6, 7–9 and 10 received scores of 1, 2, 3, 4 and 5 respectively^18^.

The facial width (pre- and postoperatively) was measured with a flexible ruler to assess the buccal space (measurement from the tragus to the angle of the mouth) and submandibular space (measurement from the tragus to the pogonion) in order to quantify the facial edema^7,8^. Facial measurements were calculated in millimeters and expressed as the simple average between the two measurements. The percentage of facial edema was calculated according to the equation: (postoperative measurement−preoperative measurement/preoperative measurement × 100)^7^.

Trismus was measured with an analog caliper between the edges of the upper and lower central incisors at maximum mouth opening. The value was calculated according to the equation: (preoperative measurement−postoperative measurement/preoperative measurement × 100)^7,8^.

How much postoperative discomfort interfered with daily activities was assessed on the 7th postoperative day using the postoperative symptoms severity questionnaire (PoSSe—Postoperative Symptom Severity Scale) with scores of 0–100^8,9,13,14^ in 7 subscales: eating, speech, local sensitivity, appearance, pain, and interference in daily activities. The higher the score, the greater the postoperative discomfort felt by the patient^13,15^.

The area of superficial necrosis (necrosis band) refers to the damage caused by the rotating instrument when in contact with the bone tissue. This would be the area of non-vital tissue observed at a histological level from the margin of the osteotomy. For this purpose, the bone biopsies performed underwent standard histological processing (fixation in 10% formaldehyde, demineralization in EDTA, dehydration in alcohol sequence, clearing with xylene and inclusion in paraffin). Microtomy with serial sections of 4 μm thickness were obtained with subsequent staining in hematoxylin and eosin (HE) for histological and histomorphometric analysis of the necrosis range and regularity of the bone edge (smooth, slightly irregular and irregular)^4,19^. After obtaining the slides, images were captured at 25 × optical microscopic magnification using a digital camera attached to the optical microscope (PrimeCam, NPlus 12, Nikon, Florida, USA) and connected to a microcomputer, for analysis using the ImageJ software (National Institutes of Health, Bethesda, USA).

New bone formation inside the alveoli was evaluated at 2 and 4 months after extraction by means of panoramic radiography taken by the same technician and with the same radiology equipment, and the images were analyzed using ImageJ software. The area of repair of the alveolar bone of the third molar was selected using the square selection of 1 cm^2^ in size. Afterwards, the images were analyzed considering the gray levels (pixel values). Pixel intensity, expressed as PI, is a measure of density, ranging from zero (black) to 255 (white). For each measurement, the histogram tool was selected to provide the mean density, standard deviation, and minimum and maximum PI for each region. Based on the tabulated density data obtained from the histogram, the CG and EG groups were compared by statistical analysis^6^.

### Statistical analysis

After tabulating the data, comparative statistics were performed using the SigmaPlot 12.0 software (Systat Software, San Jose, USA). After normality test (Shapiro–Wilk), 2-way ANOVA of repeated measures and Tukey’s post-hoc test were performed to compare pain, edema, trismus and bone repair between groups. The operative time was evaluated with independent t-test. To evaluate the PoSSe questionnaire, the range of necrosis and the mean surgical time, one-way ANOVA of repeated measures with Tukey’s post-hoc test was performed. Spearman correlation (r) was used for comparison between primary outcome variables and confounding variables. In all assessments, a significance level of 5% was used.

### Patient consent

All included patients received verbal and written information about the study and signed an informed consent agreement prior to enrolment.

### Ethical approval

The study protocol was approved by the local Ethics Committee of São Paulo State University (Unesp), School of Dentistry, Araçatuba (approval No. 49101521.2.0000.5420).

## Results

**Figure 3:**
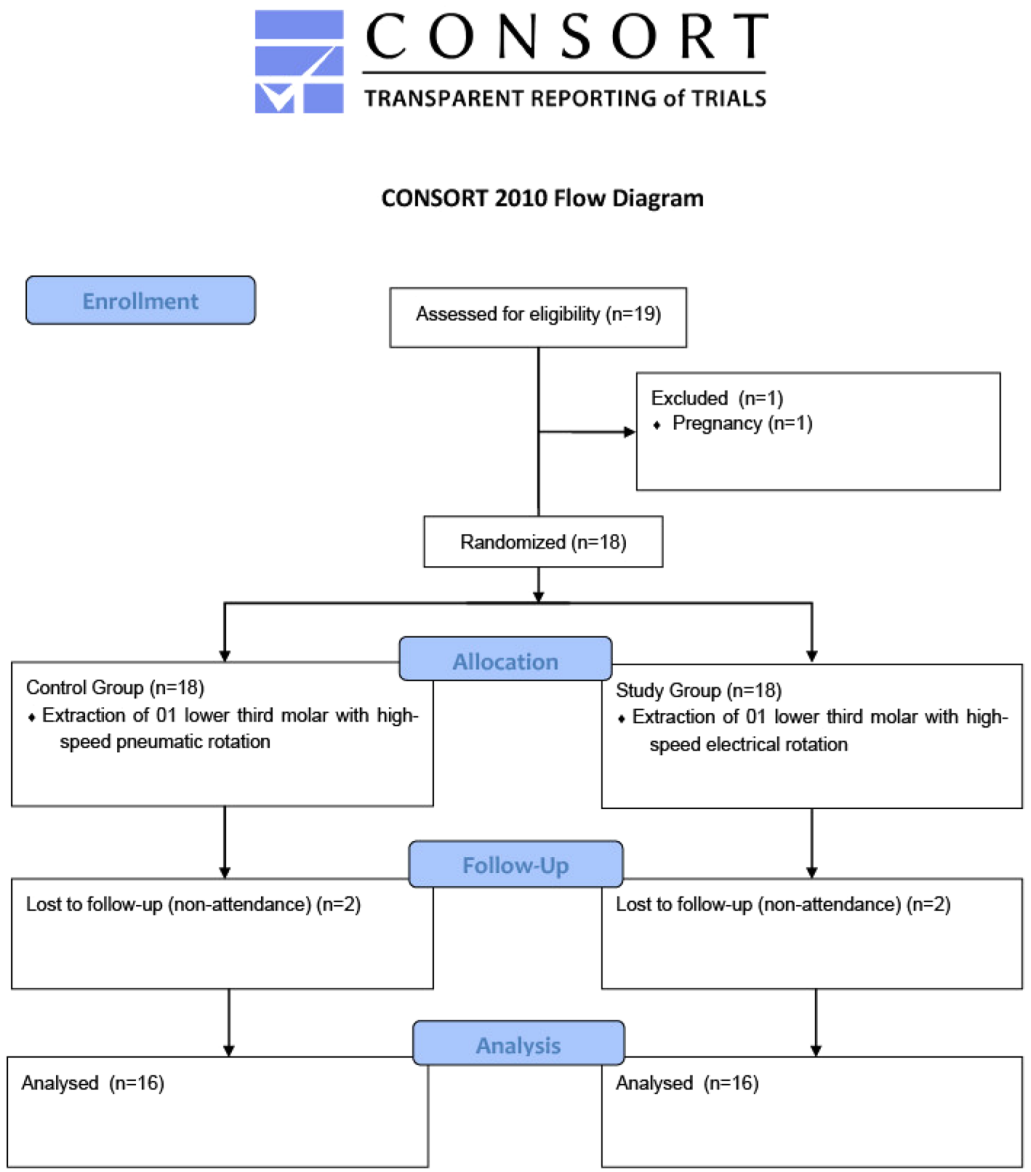
CONSORT 2010 flow chart.

Nineteen individuals were examined between July 2021 and January 2022. They were originally examined at the screening sector of the São Paulo State University (Unesp), School of Dentistry, Araçatuba, and had two mandibular third molars in similar positions identified from panoramic radiographs. One individual was excluded due to pregnancy. Eighteen individuals were included in the study—a total of 36 third molars. During follow-ups, two participants were excluded for non-attendance. Thus, the final study sample consisted of 16 participants (9 females and 7 males) aged between 15 and 43 years (mean age 24.62 years) and 32 tooth extractions (Fig. 3). Descriptive data for third molars are shown in Table 1.

**Table 1:** Analysis of the teeth included in the study.

Descriptive analysis	Total	Group	
		CG	SG
Age			
15–23	± 24,62		
Gender			
Female	9		
Male	7		
Teeth			
38	16	9	7
48	16	7	9
Winter classification			
Horizontal	14	7	7
Mesioangular	18	9	9
Pell & Gregory Classification—relationship with anterior border of ramus			
II	18	9	9
III	14	7	7
Pell & Gregory Classification—relationship to occlusal plane			
B	26	13	13
C	6	3	3

The mean duration of surgeries for the CG group was 29.13 (± 8.87) minutes, and for the SG group, 21.80 (± 7.17) minutes. The mean surgical time for the CG group was 25.16% higher than SG group and the difference was statistically significant (p = 0.0007) (Table 2).

**Table 2:** Comparative analysis of mean operative time between CG and SG groups.

Group	Surgery time (minutes)	*p*
CG	29.13 ± 8.87	0.0007*
SG	21.80 ± 7.17	

Statistical differences are represented as (*) for p < 0.05.

There were no postoperative complications of extractions in the studied groups.

### Pain

**Figure 4:**
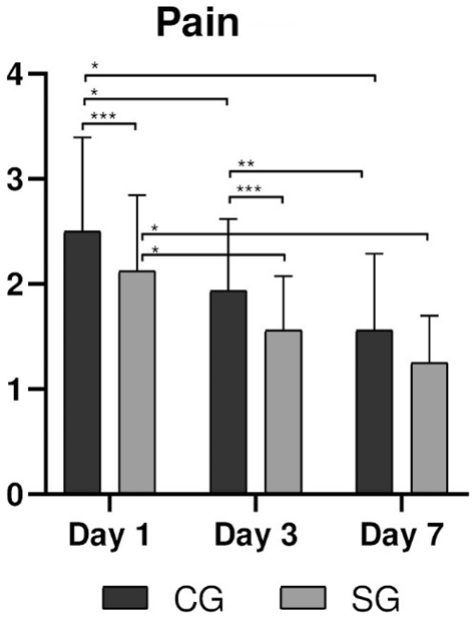
Pain levels in the CG and SG groups for periods of 1, 3, and 7 days. Statistical differences are represented as (*) for p < 0.001, (**) for p < 0.02, and (***) for p < 0.03.

Pain scores on the VAS were higher on the first day of the postoperative evaluation in both groups, with a decrease on the following evaluation days (Table 3, Fig. 4). There were significant differences (p < 0.034) between pain scores on the 1st and 3rd day, being smaller for the group SG. In the intragroup evaluation, p < 0.001 was found when comparing times 1d vs 3d and 1d vs 7d in both groups; in the evaluation between 3d vs 7d, only the CG group showed difference in means (p < 0.024).

**Table 3:** Mean and standard deviation of the pain using a visual analogue scale score, edema and trismus between the CG and SG groups in the periods of 1, 3 and 7 days.

Day	Pain			Edema			Trismus		
	CG	SG	p	CG	SG	p	CG	SG	p
1	2.5 ± 0.86	2.12 ± 0.69	0.034*	10.22 ± 7.03	7.63 ± 4.2	< 0.001*	43.81 ± 16.42	36.18 ± 17.79	0.025*
3	1.93 ± 0.65	1.56 ± 0.49	0.034*	6.24 ± 2.71	6.04 ± 4.19	*0.848*	33.41 ± 15.54	24.47 ± 10.75	0.010*
7	1.56 ± 0.70	1.25 ± 0.43	*0.075*	1.93 ± 2.71	1.61 ± 3.21	*0.210*	17.29 ± 12.06	10.03 ± 8.52	0.032*

Statistical differences are represented as (*) for p < 0.05.

Significant values are in [italics].

### Edema

**Figure 5:**
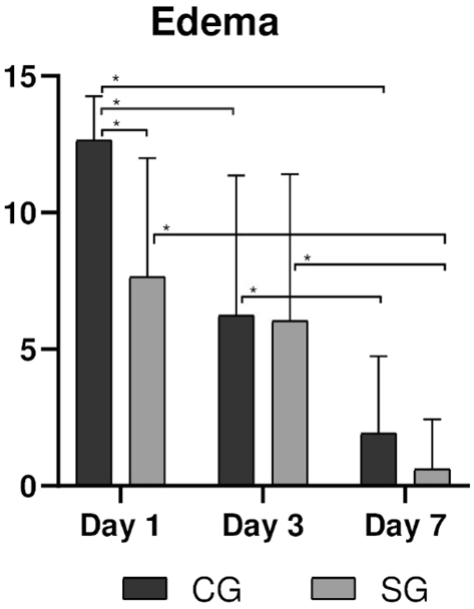
Facial edema for the CG and SG groups for periods of 1, 3, and 7 days. Statistical differences are represented as (*) for p < 0.001.

The mean percentage of edema in the three postoperative evaluation times was higher in the CG group (Table 3, Fig. 5). Overall, a higher percentage of edema was observed in the first 24 h of postoperative follow-up in CG group, with a statistical difference (p < 0.001). A gradual decline in values was observed on the 3rd and 7th day of follow-up, in both groups. Edema also reduced (p < 0.05) between 1 and 7d and 3d and 7d, for participants of both groups. Furthermore, participants of CG showed edema reduction between 1 and 3d (p < 0.001).

### Trismus

**Figure 6:**
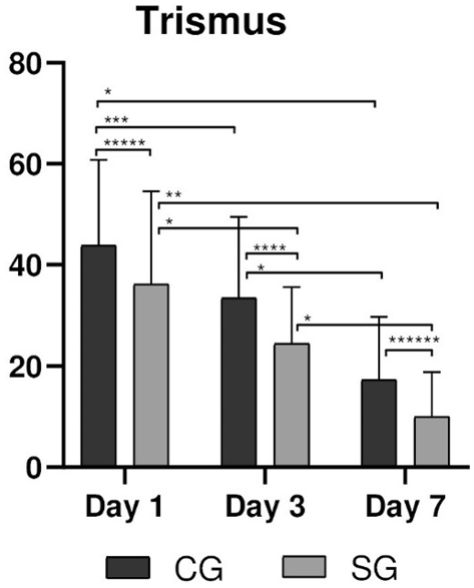
Trismus in CG and SG groups for periods of 1, 3, and 7 days. Statistical differences are represented as (*) for p < 0.01, (**) for p = 0.003, (***) for p = 0.009, (****) for p = 0.001, (*****) for p = 0.025, and (******) for p = 0.032.

The mean percentage of trismus was higher for the CG group in all evaluation periods (p < 0.05—Table 3, Fig. 6). Despite the higher values of trismus on the 1st day of the postoperative evaluation for the CG, at the end of the 7 days of evaluation the trismus was similar for both groups.

Participants of both groups had reduction of percentage of trismus when 1d was compared with all subsequent observation periods and when 3d was compared with 7d.

### Quality of life analysis

**Figure 7:**
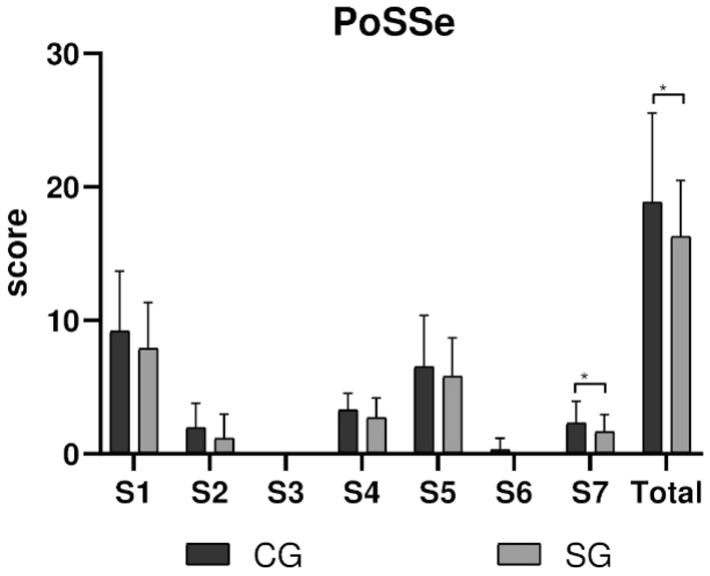
Subscales of the postoperative quality of life questionnaire using the PoSSe Scale. The statistical difference is represented with (*) for p = 0.007.

The total scores of the PoSSe scale were higher in the CG than in the SG (p = 0.007—Table 4, Fig. 7). All subscale variables were lower in the SG group. On subscale 7, which concerns the influence of symptoms on daily activities, the SG group showed significantly lower scores than CG group (p = 0.007).

**Table 4:** Mean and standard deviation of the subscales of the postoperative quality of life questionnaire by the PoSSe Scale between r CG and SG groups.

PoSSe Scale	CG	SG	p
S1. Eating	9.19 ± 4.35	7.87 ± 3.45	*0.454*
S2. Speech	1.95 ± 1.76	1.17 ± 1.79	*0.454*
S3. Sensation	0	0	*–*
S4. Appearance	3.28 ± 1.21	2.71 ± 1.47	*0.210*
S5. Pain	6.53 ± 3.7	5.82 ± 2.86	*0.804*
S6. Sickness	0.31 ± 0.82	0	*0.804*
S7. Interference with daily activities	2.3 ± 1.57	1.67 ± 1.25	0.007*
Total	23.58 ± 9.54	19.26 ± 6.93	0.007*

Statistical differences are represented as (*) for p < 0.007).

Significant values are in [italics].

### Necrosis band

**Figure 8:**
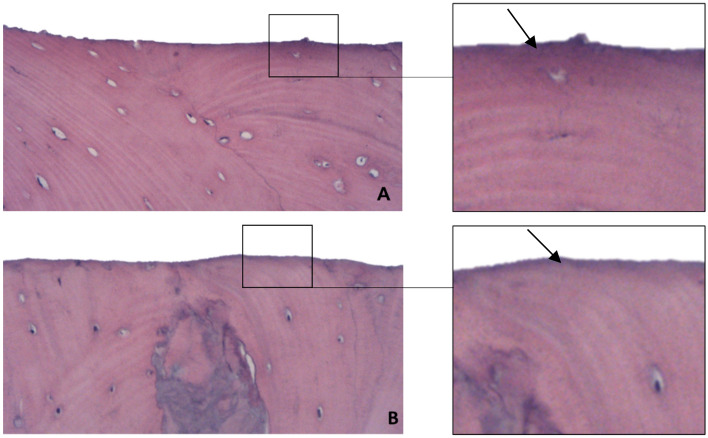
Band of necrosis (arrow) observed in the CG (**A**) and SG (**B**) groups. 40× magnification. HE staining.

The mean necrosis band was 0.013 µm for the CG group and 0.011 µm for the SG group (p = 0.298). The morphological analysis of the samples obtained after the ostectomy were similar for both groups, with smooth bone border regularity in most of the analyzed specimens. Well-organized vascularized bone tissue was observed, with visualization of a lamellar structure around the Harvesian canals, and with a well-defined and linear osteotomy line in all specimens (Fig. 8).

### Alveolar repair

Mean bone density (measured in pixels) was similar for both groups at the two evaluated periods, 2 and 4 months, (p > 0.05) for both groups (Table 5).

**Table 5:** Bone density assessed by panoramic radiography 2 and 4 months after extraction.

Group	2 months				4 months				*p*
	Mean	SD	Min	Max	Mean	SD	Min	Max	
CG	144.38	16.74	93.50	201.50	153.25	16.83	92.13	199.06	p = 0.231
SG	144.00	16.47	86.25	198.25	154.19	16.24	97.13	207.19	p = 0.143
*p*	*p* = 0.999				*p* = 0.996				

### Variables correlation

The correlation between the variables of the primary outcomes, pain, edema and trismus, and confounding factors was analyzed (Table 6). There was a statistically significant correlation between the operative time and trismus on the 1st (p = 0.01) and 7th (p = 0.03) day. The r value between 0.38 and 0.45 indicated a weak directly proportional correlation.

**Table 6:** Correlation between predictors and primary outcome pain, edema, and trismus.

Variables		Pain			Edema			Trismus		
		1d	3d	7d	1d	3d	7d	1d	3d	7d
Age	r	0.14	0.26	0.27	− 0.07	− 0.08	− 0.07	− 0.21	− 0.11	0.19
	*p*	0.59	0.31	0.28	0.67	0.64	0.67	0.24	0.53	0.28
Time	r	0.07	0.28	− 0.13	− 0.1	− 0.05	− 0.12	0.45	0.31	0.38
	*p*	0.7	0.13	0.47	0.56	0.76	0.5	0.01*	0.08	0.03*
Winters Classification	r	0.09	0.23	0.01	− 0.04	0.04	− 0.12	0.08	0.09	0.26
	*p*	0.61	0.18	0.92	0.79	0.82	0.49	0.62	0.58	0.14
Pell & Gregory—mandibular ramus	r	0	− 0.04	− 0.01	0.02	0.1	0.24	0.12	0.01	− 0.26
	*p*	0.99	0.8	0.92	0.91	0.56	0.16	0.5	0.92	0.14
Pell & Gregory—occlusal plane	r	0.15	0.19	0.28	− 0.12	− 0.23	-0.15	0.02	0.2	0
	*p*	0.4	0.28	0.11	0.5	0.19	0.4	0.88	0.27	0.96

Statistical differences are represented as (*) for p < 0.05.

There was a correlation between trismus and the post-surgical time on the 1st and 7th follow-up days. In the first 24 h, 18.75% of participants had trismus between 0-1 mm, 71.87% between 1.1 and 3 mm, and 9.37% had trismus greater than 3 mm. On the 7th day, 78.12% of participants had trismus between 0 and 1 mm, and 21.87% between 1.1 and 3 mm.

## Discussion

This study was carried out to compare the postoperative parameters of third molar extraction using pneumatic and electric high-speed turbines. Better clinical results and better patient perception of the impact of surgery on daily life and a significant reduction in operative time were observed with the use of the high-speed electric turbine.

The instruments used during the osteotomy and odontosection procedures, was effective for the technique, with different clinical outcomes being associated with each system. Cases treated with rotary systems were associated with a greater degree of edema and trismus throughout the postoperative period^8,9,20^*.* Considering that pneumatic motors are more frequently used^4,20^, comparative studies of different techniques that provide a faster cutting system and an improvement in the postoperative condition should be encouraged. Despite the lower noise production, better tactile sensation, and greater precision of the electric motors^10^, the literature is scarce in studies on their use in Oral and Maxillofacial Surgery. Comparative studies are more frequent between high and low rotation turbines and piezosurgery^4^. The use of piezoelectric for the extraction of lower third molars results in a longer clinical time of osteotomy^7,14,15,21^ and an improvement in the reported postoperative quality of life^8,9,14^ and less generation of pain, edema and trismus^7–9,14,15^. However, they require a greater financial investment and learning curve for use. Our data showed a significant reduction in surgical time (p < 0.05) and better clinical results in all primary outcomes evaluated (pain, edema and trismus) with the electric turbine. Therefore, it represents an important tool in reducing surgical trauma and optimizing symptoms that are expected after complex extractions. Although studies have reported a correlation between operative time and pain, edema and postoperative trismus^3,22^ our study found a positive correlation only with the generated trismus.

Surgical trauma initiates a complex biophysiological process that results in pain and edema^3^. Pain seems to be the main reason for the deterioration of quality of life after the extraction of third molars^12^. The pain VAS is a reliable and sensitive tool to measure self-reported pain^16,17,20^*,* which is a common and expected symptom of the repair process. Pain is most reported on the 1st postoperative day and with a significant reduction on the 7th day^22^.

The evidence found in this comparative study provided us with promising results. In the pain perception parameter, the SG group had lower pain scores (p = 0.034) on the 1st and 3rd postoperative day than the CG group, with a gradual reduction in both groups over time. This lower perception of pain reported in periods of greater inflammatory response (72 h after tooth extraction) suggests that the CG group experienced greater operative trauma and needed a longer recovery time, a hypothesis supported by the finding of a statistical difference (p < 0.05) between the 3 times of postoperative evaluation of this group. One should consider the high diversity of methodologies used to compare different systems, which report a higher pain score when comparing cutting drills and rotary systems (15,000–50,000 rpm) with ultrasonic vibrations (25–29 kHz)^3–5,8,14,15^. The comparison between two similar systems rotary (200,000 rpm and 450,000 rpm) is the novelty of this study compared to others in the literature.

The use of an electric motor for the extraction of impacted third molars also resulted in less edema and trismus. The percentage of edema and trismus generated in the first 24 postoperative hours after using pneumatic rotation was approximately 25% higher than electrical rotation, although it presents significant regression after 3 days. A significant difference (p < 0.05) was found in the percentage of trismus between the CG and SG groups on the 1st, 3rd and 7th day of the postoperative evaluation. This data, corroborated by the lowest score on the PoSSe scale for the SG group, proves that the use of a high-speed electrical turbine for the extraction of impacted third molars has better qualitative and quantitative benefits during the postoperative recovery period.

In addition to results based on the professional point of view, it is important to broaden this perspective to the patient's point of view, and to assess their perception of the treatment performed^14^. The measurement of quality of life is mainly used to assess the process and outcomes in cohort studies and randomized trials^13^. Although the influence of postoperative pain, edema and trismus on the quality of life of patients is known, many professionals ignore them or accept them as an inevitable part of the procedure, even knowing their negative impact on self-esteem and withdrawal from daily activities^13^.

The PoSSe scale, specifically used for third molar extractions, is brief and simple to fill out. It reflects the clinical severity of the postoperative symptoms and the patient's perception of the impact they have on their daily routine^8,13^. We observed excellent acceptance by patients, and they found it easy to understand the questions. A statistical difference was observed between the scores of CG and SG groups (p = 0.007), with lower scores in all subscales in the SG group, thus meaning less impact of the surgery on daily activities. The lowest scores observed in the SG group corroborate the clinical results, with the positive perception of improvement in the postoperative period by the participants, and that represents an important reduction in post-operative morbidity. This perspective is important for the professional to identify the clinical outcomes of the different techniques and to understand the patient's perception of the compared techniques.

Despite the shorter surgical time observed with the electric motor, no differences were observed in the generation of the immediate thermal necrosis band nor in local bone repair (p > 0.05) between groups. This observed similarity may be related to the efficiency of the irrigation systems, and therefore the different engine speeds (rpm) cannot be held responsible for the generation of peripheral necrosis that would delay and aggravate the local repair process. This data corroborates our hypothesis that an optimal surgical time can reduce the severity of reported postoperative signs and symptoms. The pixel density observed in both groups was similar, demonstrating that despite the worst clinical parameters observed with the pneumatic motor, there was no influence on local repair after 2 and 4 months. Thus, when using pneumatic rotation, a greater inflammatory condition can be expected without precluding the long-term repair. In a clinical study comparing the piezoelectric with the rotary system, the pneumatic turbine showed no difference in bone repair despite its unfavorable clinical results^23^.

One of the limitations of this study was the impossibility of double blinding due to the physical differences of the engines. However, evaluators were blinded to the treatments. The use of randomization contributed to the reduction of research bias, allowing the observation of the same patient's perception of the different proposed surgical protocols. The research field of third molar extractions with an electric rotary system should be considered for future research in the field of Oral and Maxillofacial Surgery.

## Conclusion

The use of a high-speed electric turbine showed a shorter operative time, less pain, edema and trismus, and a better quality of life after the extraction of impacted third molars.

## Data Availability

The study protocol and all data are available from the corresponding author on reasonable request.
